# A scoping review on the methodological and reporting quality of scoping reviews in China

**DOI:** 10.1186/s12874-024-02172-y

**Published:** 2024-02-22

**Authors:** Xinyu Xue, Xintong Tang, Shanshan Liu, Ting Yu, Zhonglan Chen, Ningsu Chen, Jiajie Yu

**Affiliations:** 1 Department of Clinical Nutrition, West China Hospital, Sichuan University, Chengdu, 610041 China; 2 Chinese Evidence-based Medicine Center, West China Hospital, Sichuan University, Chengdu, 610041 China; 3 Evidence-based Nursing Center, West China School of Nursing, West China Hospital, Sichuan University, Chengdu, 610041 China

**Keywords:** Scoping review, Literature review, Methodology, China

## Abstract

**Background:**

Scoping reviews have emerged as a valuable method for synthesizing emerging evidence, providing a comprehensive contextual overview, and influencing policy and practice developments. The objective of this study is to provide an overview of scoping reviews conducted in Chinese academic institutions over the last decades.

**Method:**

We conducted a comprehensive search of nine databases and six grey literature databases for scoping reviews conducted in Chinese academic institutions. The reporting quality of the included reviews was assessed using the Preferred Reporting Items for PRISMA-ScR checklist. We performed both quantitative and qualitative analyses, examining the conduct of the scoping reviews and exploring the breadth of research topics covered. We used Chi-squared and Wilcoxon rank-sum tests to compare methodological issues and reporting quality in English and Chinese-language reviews.

**Results:**

A total of 392 reviews published between 2013 and 2022 were included, 238 English-reported reviews and 154 Chinese-reported reviews, respectively. The primary purposes of these reviews were to map and summarize the evidence, with a particular focus on health and nursing topics. 98.7% of reviews explicitly used the term “scoping review”, and the Arksey and O’Malley framework was the most frequently cited framework. Thirty-five English-reported scoping reviews provided a protocol for scoping review. PubMed was the most common source in English-reported reviews and CNKI in Chinese-reported reviews. Reviews published in English were more likely to search the grey literature (*P* = 0.005), consult information specialists (*P* < 0.001) and conduct an updated search (*P* = 0.012) than those in Chinese. Reviews published in English had a significantly high score compared to those published in Chinese (16 vs. 14; *P* < 0.001). The reporting rates in English-reported reviews were higher than those in Chinese reviews for seven items, but lower for structured summary (*P* < 0.001), eligibility criteria (*P* < 0.001), data charting process (*P* = 0.009) and data items (*P* = 0.015).

**Conclusion:**

There has been a significant increase in the number of scoping reviews conducted in Chinese academic institutions each year since 2020. While the research topics covered are diverse, the overall reporting quality of these reviews is need to be improved. And there is a need for greater standardization in the conduct of scoping reviews in Chinese academic institutions.

**Supplementary Information:**

The online version contains supplementary material available at 10.1186/s12874-024-02172-y.

## Introduction

Along with the increased production of primary research, the conduct and publication of evidence synthesis have also increased over time [1]. To address various questions for policymakers and other stakeholders, different types of reviews have emerged [2], with 48 types of reviews identified by 2019 [3]. One of the review types is the “scoping review”, also known as a “scoping study”, or “mapping review” [4]. Scoping reviews are a valuable approach to synthesizing emerging evidence, providing a comprehensive overview of the context and the potential to influence policy and practice developments [5, 6]. Scoping reviews have been widely conducted in various fields, including health, technology and social sciences over the past decades [7].

Scoping reviews follow similar processes to systematic reviews in terms of identifying and analyzing relevant literature on a specific topic [8]. Scoping review characteristically involves the development, assimilation, and synthesis of a broad base of evidence derived from a diverse range of published research and grey literature research [9]. It aims to clarify the key concepts and characteristics that underpin a research area, determine a precise volume of literature and studies available, or can be a precursor to a systematic review [10]. Unlike systematic reviews, scoping reviews do not require a quality assessment of individual studies or the integration of evidence from different studies [11]. While a scoping study requires a framework to investigate existing literature, it does not involve assessing the weight of evidence for particular interventions or policies [12].

Scoping review is widely used to answer board research questions now. The concept was initially proposed by Mays in 2001 [13] and later used by Arksey and O’Malley, who provided the first guidance on conducting scoping review in 2005 [14]. In 2014, the JBI and JBI collaboration published their guidance on scoping review [15] and updated it in 2020 [16, 17]. As a result, the publication of scoping reviews has significantly increased. To improve the methodological and reporting quality of scoping reviews, the Preferred Reporting Items for Systematic Reviews extension for Scoping Review [18] (PRISMA-ScR) was published in 2018.

The research community involved in scoping reviews, mainly from Canada, the United States, the United Kingdom, and Australia, shows a steady increase in literature and maintains a relatively high growth rate [4]. China is currently in the early stages of introducing and familiarizing itself with the scoping review methodology, with few documented examples of its practical application in the field of medicine [19]. Additionally, some review articles in China, although conceptually similar to scoping reviews, did not follow the standardized methodology and therefore could not be classified as scoping reviews [20, 21]. This may be attributed to a lack of awareness and a delay in the adoption of scoping reviews by Chinese academic authors.

In light of the above situation and the growing utilization of scoping reviews in China, our study aims to (1) examine volume, scope and distribution of scoping reviews conducted in Chinese academic institutions; (2) summarize the purpose, topics, and methodological issues in these scoping reviews; (3) explore the extent to which scoping review adheres to reporting guidelines.

## Methods and analysis

We used Arksey and O’Malley’s framework [14] on scoping review to guide our study. This protocol was registered on the open science framework (https://osf.io/f9u6q/).

### Identify the research questions

Our research questions were as follows.

1) What is the volume, scope and distribution of scoping reviews conducted in Chinese academic institutions?

2) What are the purposes, topics and methodological issues in the included scoping reviews?

3) To what extent do these scoping reviews adhere to the PRISMA ScR reporting guidelines?

### Eligibility criteria

We included all scoping reviews that met the following criteria: (1) utilized a scoping review of the literature approach with a description of the synthesis method used and (2) focused on the field of health/medicine. We excluded studies that (1) did not synthesize literature, such as complete scoping of surveillance or administrative databases; (2) primarily described scoping review methods or guidelines and (3) full text was not available. We defined scoping reviews conducted in Chinese academic institutions as the corresponding author’s affiliations located in mainland China, Hong Kong, Macau or Taiwan.

### Identify relevant studies

We conducted a comprehensive literature search from the inception of the following five English electronic databases and four Chinese databases until Dec 2021, with an update to Dec 2022: PubMed, EMBASE, EBSCO, Web of Science, The Cochrane Library, SinoMed, VIP Chinese periodical Service, Wanfang Data Knowledge Service, and China National Knowledge Infrastructure (CNKI). Grey literature (e.g. thesis and dissertation, newspaper, conference paper) and some web search engines (e.g. Google, Google Scholar, Baidu, Baidu Scholar) were also searched. The search strategy was not restricted by study design and an expert information specialist collaborated with the research team. The search strategies were listed in Appendix 1 We also cross-checked the included studies and references of a relevant scoping review.

### Study selection

Inter-rater agreement for study inclusion was calculated using percent agreement. If the agreement exceed 75% among the team members, we proceeded to the next stage. All title and abstract screening and full-text screening were performed independently by at least two review authors (Xue XY, Tang XT, Liu SS, Yu T) using a pre-defined form. Any discrepancies were resolved through consensus or involving a third reviewer (Yu JJ) when necessary.

### Charting the data

The following general information was collected from each eligible study: published year, regions, affiliations, journal name, study population, number of studies included, and funding source. We also collected details on the purpose of the scoping review (e.g., identified evidence gaps, future research opportunities, implications for policy or practice), as well as the topic addressed. We collected methodological information including the definition of “scoping review”, utilization of established methodological guidance (e.g., Arksey and O’Malley, Levac, Joanna Briggs Institute, or others), protocol and registration, research question, inclusion criteria, eligible study design (e.g., primary studies, secondary studies, both, or others), search strategy, databases searched, additional search resources (e.g. explore breadth/extent of evidence, grey literature, consulted experts, crosscheck references), title and abstract screening, full-text screening, pre-defined charting form, flow diagram, result presentation (tables and/or diagrams), the implication for research and practice [22].

We assessed the reporting quality of eligible scoping reviews using the PRISMA-ScR checklist [18], which includes 20 items and 2 optional items for critical appraisal of individual studies. Each item was determined with the option of “yes” or “no”, allocating 1 point if the study met the requirement for a specific item and 0 if not. A total score ranging from 0 to 22 was developed.

Data extraction and reporting quality from each eligible study were conducted by four reviewers, a pilot study was performed before formal extraction, and the interrater agreement percentage needed to be > 75%. Discrepancies were resolved by consensus or the involvement of a third reviewer.

### Collating and summarizing results

We conducted both quantitative and qualitative analyses on the scoping reviewsThe quantitative analysis involved examining the distribution of reviews, methodological issues, and reporting quality. For quantitative analysis, frequencies and proportions were calculated for the categorical variables and mean (SD), median (range) or median (IQR) were used to analyze the continuous variables. Word clouds were generated using the online program WordClouds to visualize the synthesis topics (Zygomatic, 2022) (https://www.wordclouds.com).

We performed a comparative analysis of methodological issues and reporting quality between eligible scoping reviews published in English and Chinese. Either X^2^ or Fisher’s exact tests were used for the analysis of categorical variables and the Wilcoxon rank-sum test was used for continuous data with a non-normal distribution. For qualitative analysis, two reviewers independently categorized the key components, and the results were subsequently discussed by the research team.

## Results

### Search and selection of scoping review

**Fig. 1:**
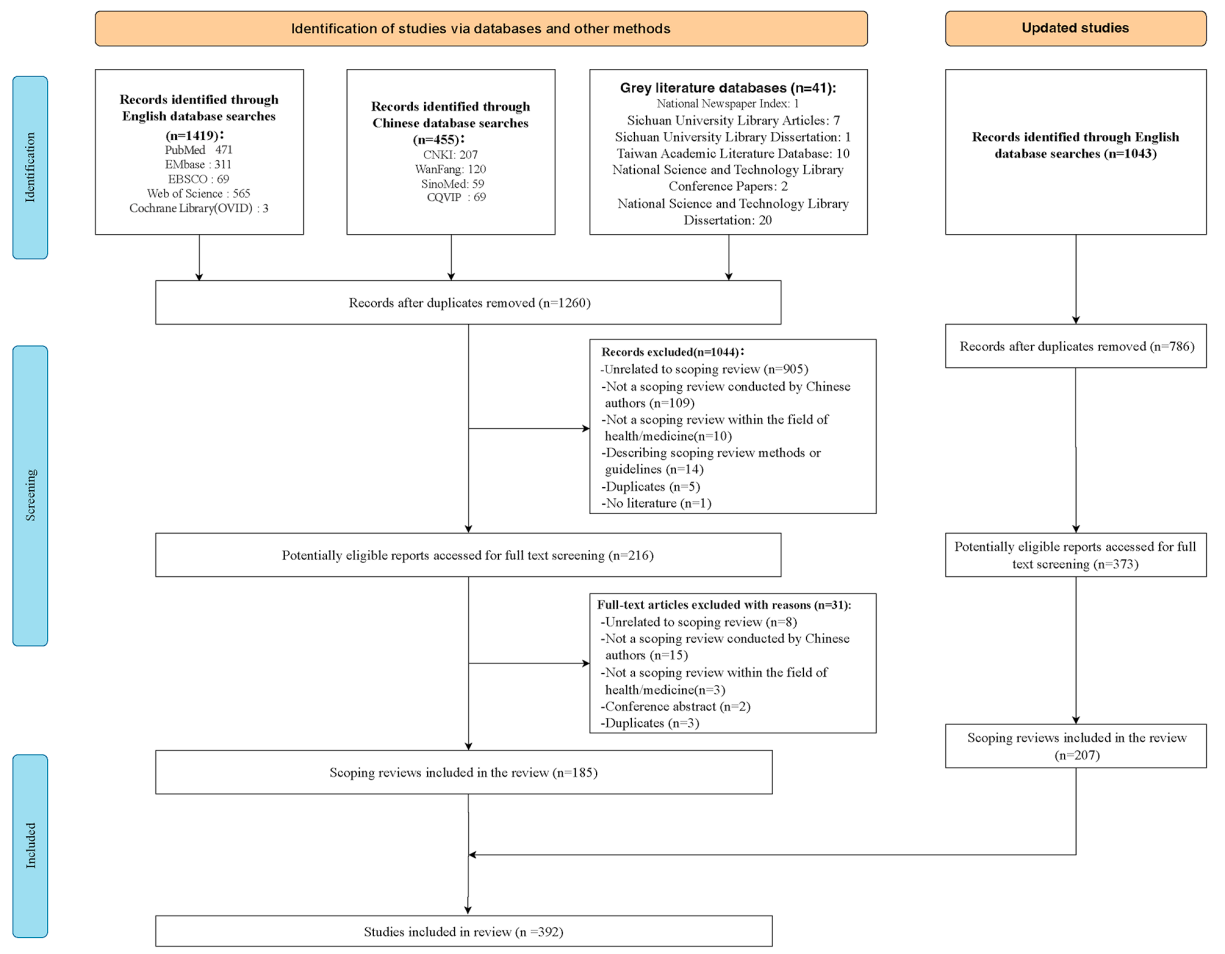
Study selection Details the flow of information through the different phases of the review; map out the number of records identified, included and excluded, and the reasons for their exclusion

A total of 2958 citations relevant to scoping review were searched, and 2046 studies were included for screening after duplication. After reading 589 potentially relevant full-text papers, 392 articles were finally included (Fig. 1). The interrater agreements among the four reviewers were good, with agreement rates of 92.2% at the title and abstract screening, 94.8% at the full-text reading, and 95.3% at the table extraction.

### Study characteristics of including scoping reviews

**Fig. 2:**
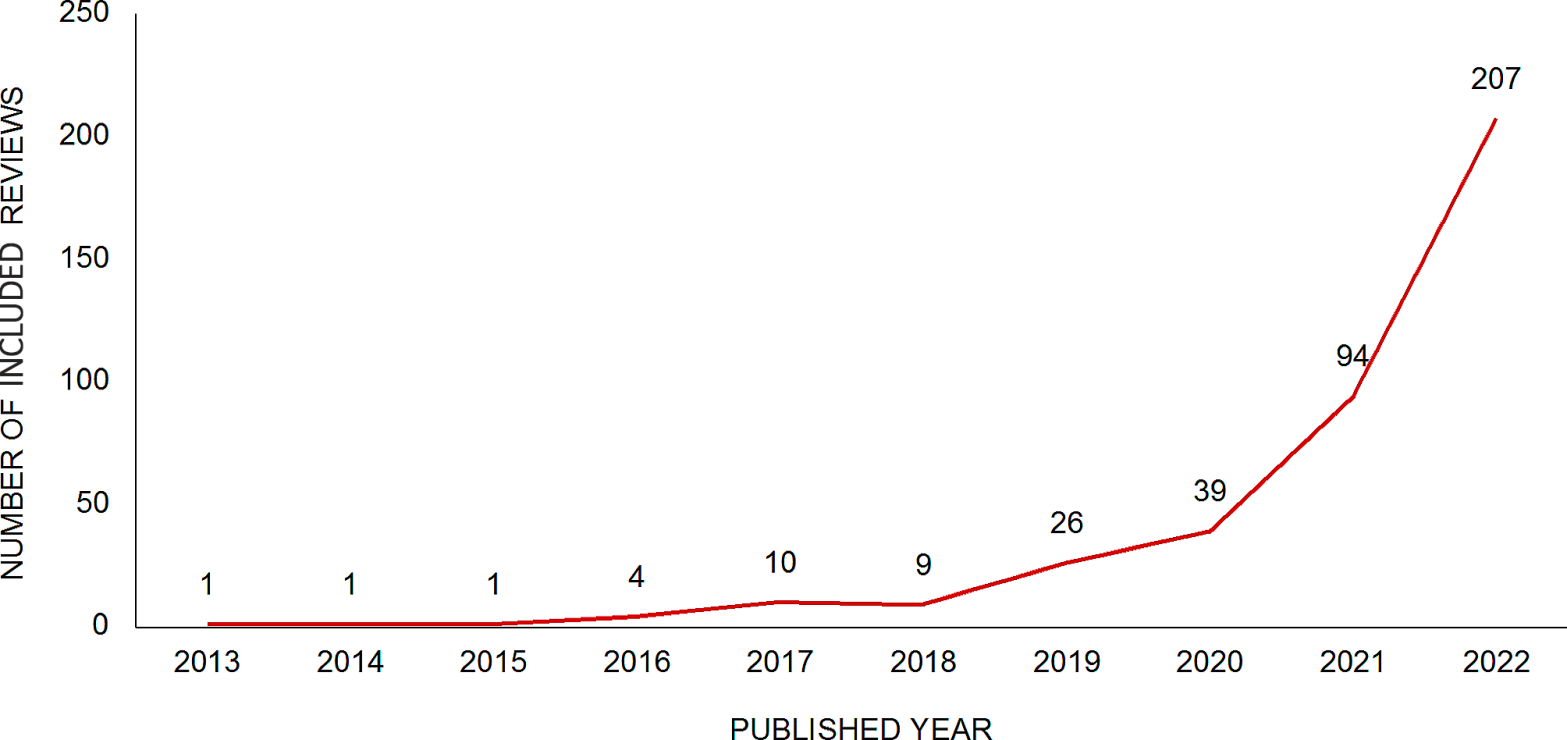
Published year The annual number of scoping reviews conducted by Chinese academic institutions is visually represented in the form of a line graph

The scoping reviews included in our study were published between 2013 and 2022, with a significant majority (*n* = 340, 86.7%) published after 2020 (Fig. 2). The median number of studies included in these scoping reviews was 29 (Range: 5-6430). Among the included reviews, 238 (60.7%) were reported in English. A total of 217 journals were involved in publishing the scoping review, with the Chinese Journal of Nursing (*n* = 14, 3.6%) and the International Journal of Environmental Research and Public Health (*n* = 10, 2.6%) being the most commonly published Chinese and English journals, respectively (Table 1). Most of the reviews were conducted in Beijing (*n* = 66, 16.8%), Hong Kong (*n* = 59, 15.1%), and Shanghai (*n* = 44, 11.2%) (Appendix 2). The reviews originated from 166 institutions, primarily universities and hospitals (Table 1). 274 (69.9%) received funding support, with majority (98.5%) being publicly sponsored. Notably, the distribution of these characteristics was different between Chinese reviews and English reviews (Table 1).

**Table 1:** Study characteristics of included reviews

	Total (*n* = 392, %)	English (*n* = 238, %)	Chinese (*n* = 154, %)	***P*** ^†^
***Published year***				0.630^**‡**^
2013–2018	26 (6.6)	17 (7.1)	9 (5.8)	
2019	26 (6.6)	18 (7.6)	8 (5.2)	
2020	39 (9.9)	25 (10.5)	14 (9.1)	
2021	94 (24.0)	55 (23.1)	39 (25.3)	
2022	207 (52.8)	57 (71.7)	34 (54.5)	
***No. of studies included*** ^*^	29(5-6430)	35(5-6430)	24(6-3112)	0.004^**‡**^
***Journal name***				< 0.001
Chinese Journal of Nursing	14 (3.6)	0 (0)	14 (9.1)	
Chinese Nursing Management	11(2.8)	0(0)	11(7.1)	
Journal of Nursing Science	11(2.8)	0(0)	11(7.1)	
International Journal of Environmental Research and Public Health	10 (2.6)	10 (4.2)	0 (0)	
Infectious Diseases of Poverty	9 (2.3)	9 (3.8)	0 (0)	
Other	337(86.0)	219(92.0)	118(76.6)	
***Regions***				< 0.001
Beijing	66 (16.8)	27 (11.3)	39 (25.3)	
Hong Kong	59 (15.1)	59 (24.8)	0 (0)	
Shanghai	44 (11.2)	19 (8.0)	25 (16.2)	
Guangdong	30(7.7)	19(8.0)	11(7.1)	
Hubei	28(7.1)	14(5.9)	14(9.1)	
Other	165(42.1)	100(42.0)	65(42.2)	
***Affiliation***				< 0.001
Hong Kong Polytechnic University	21 (5.4)	21 (8.8)	0 (0)	
Huazhong University of Science and Technology	17(4.3)	11(4.6)	6(3.9)	
China Academy of Chinese Medical Sciences	16 (4.1)	8 (3.4)	8 (5.2)	
Chinese University of Hong Kong	14 (3.6)	14 (5.9)	0 (0)	
University of Hong Kong	14(3.6)	14(5.9)	0 (0)	
Other	310(79.1)	170(71.4)	140(90.9)	
***Funding***				< 0.001
Publicly sponsored	270 (68.9)	158 (66.4)	112 (72.7)	
Industry-sponsored	4 (1.0)	4 (1.7)	0 (0)	
Non-sponsored	26 (6.6)	26 (10.9)	0 (0)	
Not reported	92(23.5)	50(21.0)	42(27.3)	

Values in parentheses are percentages unless indicated otherwise; *values are median(range). n, number of studies. ^**†**^ X^2^ test, except ^**‡**^Wilcoxon rank-sum test

**Fig. 3:**
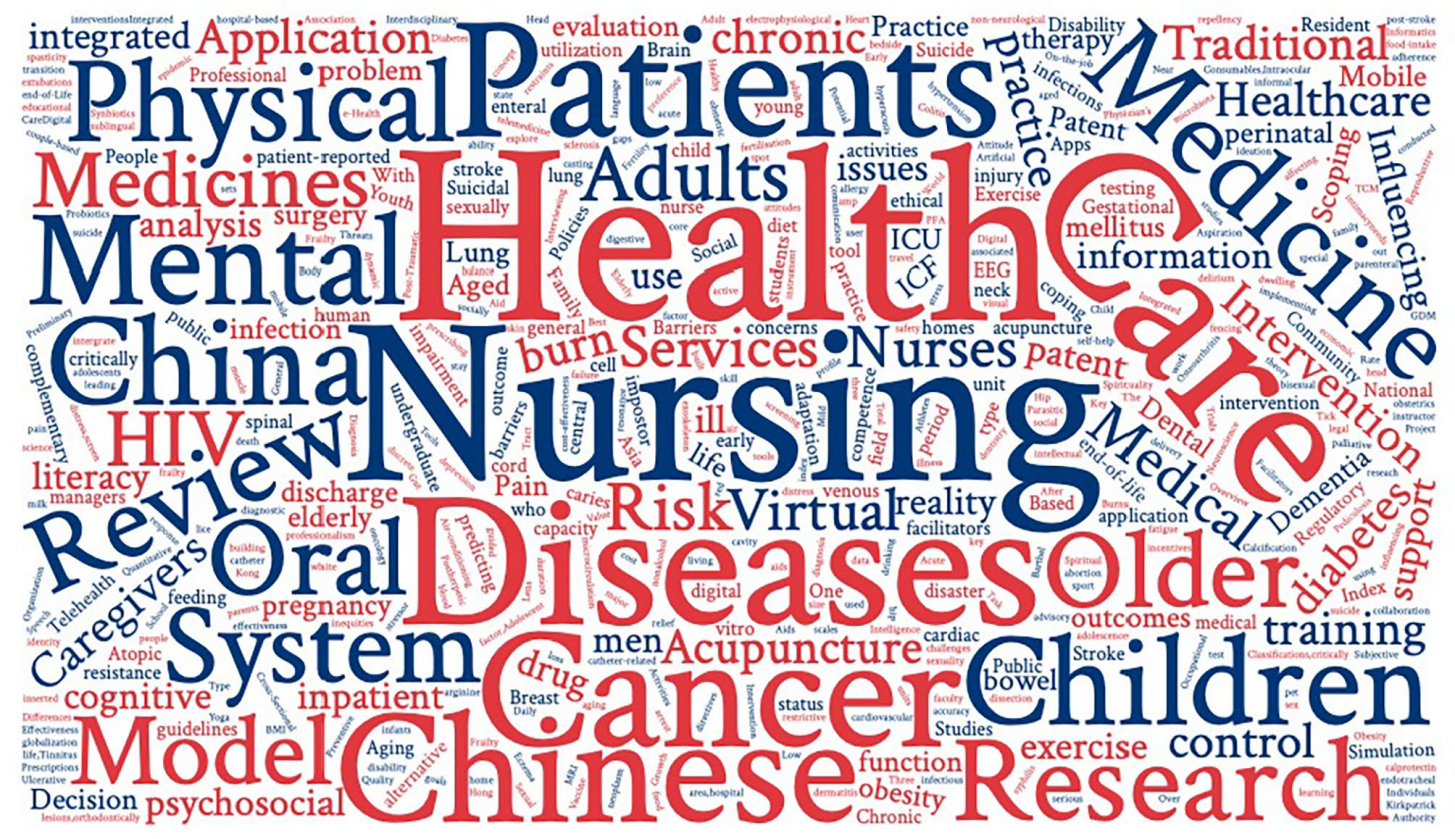
Word cloud of topics The most common topic in the 392 scoping reviews is displayed, with the size of the topics in the word cloud corresponding to the frequency of their show

The study population for the included scoping review primarily consisted of patients (*n* = 200, 51.0%) and healthcare professionals (*n* = 60, 15.3%) (Appendix 3). The main purposes of the reviews were to map and summarize existing evidence (*n* = 230, 58.7%), followed by to identify and/or address knowledge gaps (*n* = 111, 28.3%) (Appendix 4). The topics covered in the reviews were diverse, with health and nursing being the common topics (Fig. 3).

### Method characteristics of including scoping reviews

Of the 392 scoping reviews, the majority (387, 98.7%) explicitly identified themselves as “scoping review”, while 122 (31.1%) provided a definition of the term ‘scoping review’. The most frequently cited framework for conducting scoping review was Arksey and O’Malley framework (2005), referenced in 157 reviews (40.1%). The research question was clearly stated in 39.8% of the reviews, while 75.8% clearly reported their inclusion criteria.

Thirty-four (8.7%) English-reported scoping reviews provided a protocol of scoping review, and 20 (5.1%) were registered on the Open Science Framework (OSF). The majority of reviews (369, 94.1%) searched more than one database, with PubMed (*n* = 184, 77.3%) being the most common source for English-language reviews and CNKI (*n* = 137, 89.0%) for Chinese-language reviews. In terms of additional search, English-reported reviews were more likely to search the grey literature (70 vs. 39, *P* = 0.005), consult information specialists (18 vs. 3, *P* < 0.001) and conducted an updated search (16 vs. 2, *P* = 0.012) compared to Chinese-reported reviews (Table 2).

**Table 2:** Method characteristics of included reviews

		Total (*n* = 392, %)	English (*n* = 238, %)	Chinese (*n* = 154, %)	***P*** ^†^
Protocol and review design	**Protocol**				< 0.001
	Registered	30(7.7)	30(12.6)	0(0)	
	Published	4(1.0)	4(1.7)	0(0)	
	**Research question**				0.033^**‡**^
	Clearly reported	156(39.8)	109(45.8)	47(30.5)	
	Simply reported	118(30.1)	60(25.2)	58(37.7)	
	Inferred	115(29.3)	66(27.7)	49(31.8)	
	Not reported	3(0.8)	3(1.3)	0(0)	
	**Inclusion criteria**				0.174^**‡**^
	Clearly reported	297(75.8)	179(75.2)	118(76.6)	
	Simply reported	75(19.1)	44(18.5)	31(20.1)	
	Inferred	17(4.3)	15(6.3)	2(1.3)	
	Not reported	14(3.6)	11(4.6)	3(1.9)	
	**Eligible study design**				0.697
	All study designs	13(3.3)	7(2.9)	6(3.9)	
	Primary and secondary research	57(14.5)	38(16.0)	19(12.3)	
	Primary research only	201(51.3)	120(50.4)	81(52.6)	
	Secondary research only	7(1.8)	3(1.3)	4(2.6)	
	Not reported	114(29.1)	70(29.4)	44(28.6)	
Identifying relevant studies	**Search strategy**				0.009^**‡**^
	Clearly reported	200(51.0)	136(57.1)	64(41.6)	
	Keywords only	178(45.4)	91(38.2)	87(56.5)	
	Not reported	14(3.6)	11(4.6)	3(1.9)	
	**Databases searched**				0.002^**‡**^
	Searched > 1 database	369(94.1)	217(91.2)	152(98.7)	
	Searched only 1 database	21(5.4)	20(8.4)	1(0.6)	
	Not reported	2(0.5)	1(0.4)	1(0.6)	
	**Additional search strategy**				
	Grey literature searched	109(27.8)	70(29.4)	39(25.3)	0.005
	Google Scholar	43(11.0)	38(16.0)	5(3.2)	
	OpenGrey	18(4.6)	13(5.5)	5(3.2)	
	Google search	11(2.8)	8(3.4)	3(1.9)	
	ProQuest dissertations	7(1.8)	5(2.1)	2(1.3)	
	National drug catalogs	6(1.5)	0(0)	6(3.9)	
	Consulted information specialist	21(5.4)	18(7.6)	3(1.9)	< 0.001
	Consulted content experts	14(3.6)	11(4.6)	3(1.9)	0.164
	Manual searching	115(29.3)	90(37.8)	25(16.2)	< 0.001
	Updated search	18(4.6)	16(6.7)	2(1.3)	0.012
	**Limits applied**				
	Limited by study design	268(68.4)	163(68.5)	105(68.2)	0.798
	Limited by date	344(87.8)	198(83.2)	146(94.8)	0.003
	Limited by language	278(70.9)	172(72.3)	106(68.8)	0.173
	**Title and abstract screening details**				0.154
	≥ 2 independent reviewers	311(79.3)	182(76.5)	129(83.8)	
	1 reviewer + 2 verifiers	1(0.3)	1(0.4)	0(0)	
	1 reviewer + 1 verifier	4(1.0)	4(1.7)	0(0)	
	1 reviewer only	8(2.0)	7(2.9)	1(0.6)	
	Done but unclear reviewers	51(13.0)	35(14.7)	16(10.4)	
	Not reported	17(4.3)	9(3.8)	8(5.2)	
	**Full-text screening details**				0.238
	≥ 2 independent reviewers	312(79.6)	183(76.9)	129(83.8)	
	1 reviewer + 2 verifiers	1(0.3)	1(0.4)	0(0)	
	1 reviewer + 1 verifier	4(1.0)	4(1.7)	0(0)	
	1 reviewer only	5(1.3)	4(1.7)	1(0.6)	
	Done but unclear reviewers	53(13.5)	37(15.5)	16(10.4)	
	Not reported	17(4.3)	9(3.8)	8(5.2)	
Data abstraction	**Pre-defined form**	119(30.4)	65(27.3)	54(35.1)	0.103
	**Data charting details**	304(77.6)	167(70.2)	137(89.0)	< 0.001
	**Data charting process**				< 0.001
	≥ 2 independent reviewers	226(57.7)	120(50.4)	106(68.8)	
	1 reviewer + 1 verifier	14(3.6)	13(5.5)	1(0.6)	
	1 reviewer only	5(1.3)	3(1.3)	2(1.3)	
	Done but unclear reviewers	113(28.8)	74(31.1)	39(25.3)	
	Not reported	34(8.7)	28(11.8)	6(3.9)	
Quality appraisal	**Quality appraisal**	56(14.3)	40(16.8)	16(10.4)	< 0.001
	Cochrane ROB tool	8(2.0)	7(2.9)	1(0.6)	
	STROBE*	5(1.3)	4(1.7)	1(0.6)	
	AMSTAR-2	5(1.3)	5(2.1)	0(0)	
Results	**Synthesis**				
	Meta-analysis conducted	6(1.5)	5(2.1)	1(0.6)	0.243
	Formal qualitative analysis	162(41.3)	135(56.7)	27(17.5)	< 0.001
	**Reporting**				
	Flow diagram	302(77.0)	212(89.1)	90(58.4)	< 0.001
	Data in tabular format	349(89.0)	209(87.8)	140(90.9)	0.338
	Data in graphical format	122(31.1)	97(40.8)	25(16.2)	< 0.001
Discussion	Recommended policy/practice	254(64.8)	154(64.7)	100(64.9)	0.963
	Recommended future research	291 (74.2)	172(72.3)	119(77.3)	0.269
	Recommended systematic review	25(6.4)	18(7.6)	7(4.5)	0.472
	Limitations	243(62.0)	193(81.1)	50(32.5)	< 0.001
	Strengths	90(23.0)	68(28.6)	22(14.3)	0.001

* STROBE: Strengthening the reporting of observational studies in epidemiology

n: number of studies

^**†**^ X^2^ test, except ^**‡**^Wilcoxon rank-sum test

Approximately 80% of reviews screened title/abstract and full-text articles with more than two reviewers. A predefined abstraction form was used in 30.4% of the reviews and data extraction involved more than two reviewers in 57.7% of reviews. Among the included reviews, 56 reviews (14.3%) assessed the quality of the studies and six reviews (1.5%) conducted a meta-analyses. More than 75% of included reviews provided a study flow chart, while the difference was significant between reviews in English language and Chinese (212 vs. 90, *P* < 0.001). Additionally, nearly 90% of the reviews presented their results in tabular form, while 31.1% used graphical representation. In terms of discussion, a higher proportion of scoping reviews published in English journals compared to Chinese journals mentioned the limitations of their studies (193 vs. 50, *P* < 0.001) as well as their strengths (68 vs. 22, *P* = 0.001) (Table 2).

### Reporting quality of including scoping reviews

**Fig. 4:**
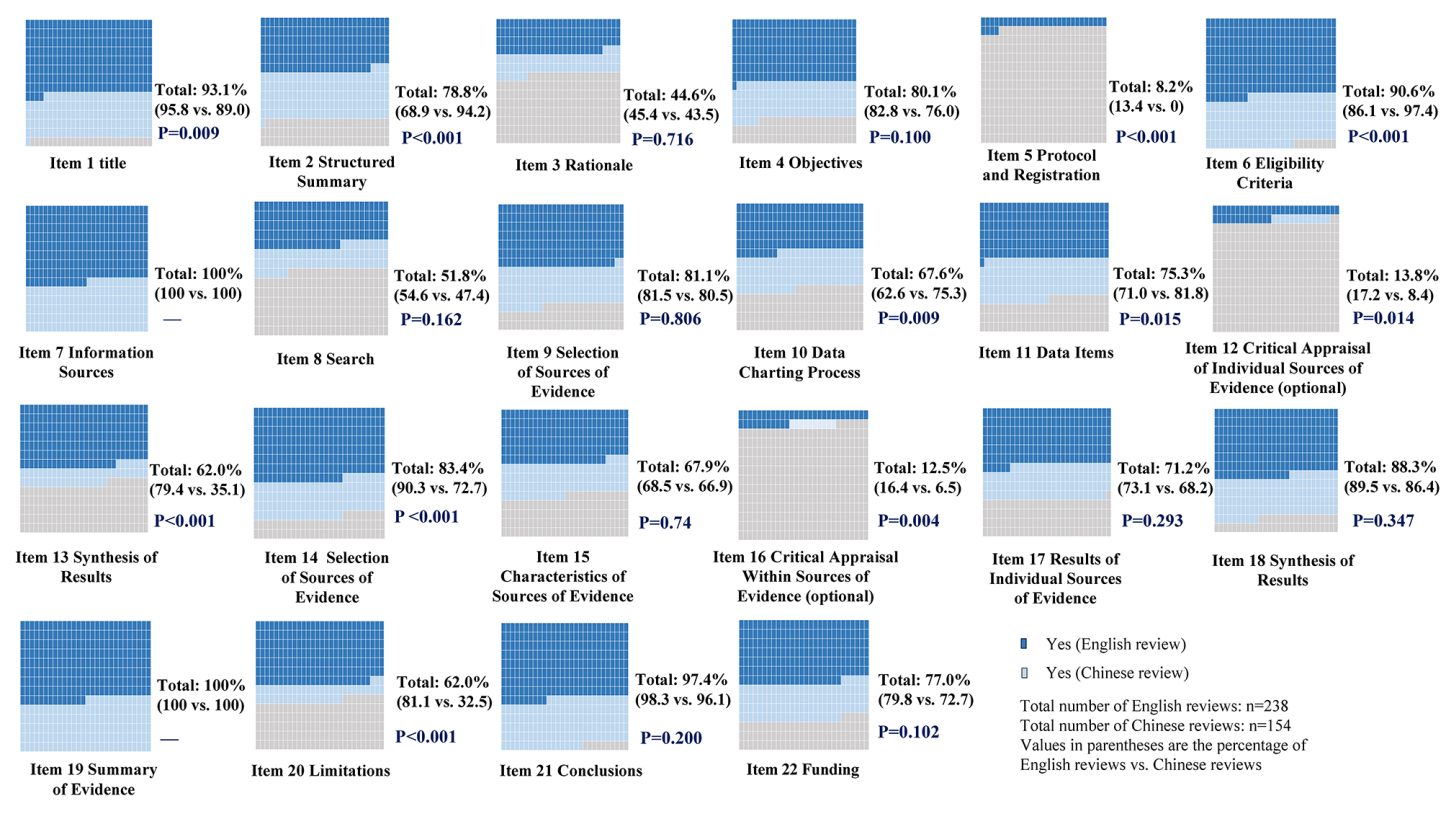
Reporting quality of including reviews

Of the 22 items, six (27.3%) were adequately reported: identification of the report as a scoping review in the title (93.1%), statement of eligibility criteria for included evidence (90.6%), description of all sources of information used in the search (100.0%), process for synthesizing the results (88.3%), methods of summarizing the evidence (100.0%) and presentation of a conclusion (97.4%). On the other hand, four items were reported less than 50% of the scoping reviews, including description of the rationale for the review (44.6%), accessibility of a protocol and registration information (8.2%), critical appraisal of individual sources of evidence (13.8%), and critical appraisal within sources of evidence (12.5%) (Fig. 4).

The median score on the PRISMA-ScR checklist was 15 (i.q.r 13–17) and the reviews published in English had significantly higher score than those in Chinese (16 [14–18] vs. 14 [13–16]; *P* < 0.001). Scoping reviews published in English were more likely than those in Chinese to report the title (95.8% versus 89.0%; *P* = 0.009), protocol and registration (13.4% versus 0.0%; *P* < 0.001), critical appraisal of individual sources of evidence (17.2% versus 8.4%; *P* = 0.014), synthesis of results (79.4% versus 35.1%; *P* < 0.001), selection of sources of evidence (90.3% versus 72.7%; *P* < 0.001), critical appraisal within sources of evidence (16.4% versus 6.5%; *P* = 0.004) and limitations (81.1% versus 32.5%; *P* < 0.001). On the other hand, Chinese reviews preferred to report structured summaries (94.2% versus 68.9%; *P* < 0.001), eligibility criteria (97.4% versus 86.1%; *P* < 0.001), data charting process (75.3% versus 62.6%; *P* = 0.009), data items (81.8% versus 71.0%; *P* = 0.015) compared to English reviews (Fig. 4).

## Discussion

We conducted a comprehensive scoping review of 392 Chinese-authored scoping reviews over the last decade. Our findings revealed a significant increase in the number of scoping reviews in China, particularly since 2020, with 207 scoping reviews published in the past year alone, accounting for approximately half of the total number published in the previous decade. The increase in the number of scoping review publications after 2020 could be attributed to several possible reasons. First, the COVID-19 pandemic in 2020 may have led to a broader literature review in the fields of health sciences and medicine to better understand and respond to the crisis. Second, scoping reviews have gained recognition within the academic and research community, leading to an increasing number of studies adopting scoping reviews for literature synthesis. Third, as research fields continue to evolve and expand, study topics become increasingly complex. Scoping reviews offer a flexible approach covering a wide range of literature, helping researchers to gain a more comprehensive understanding of the current state of research. Finally, some academic institutions and publishers may actively promote the use of scoping review methodology.

The majority of scoping reviews in our studies were conducted in hospitals and universities, with nursing and health being the predominant topics. This trend can be attributed to the education and dissemination efforts of organizations such as Joanna Briggs Institute (JBI) and its collaboration. Additionally, most studies provided a clear definition of scoping reviews and followed the framework by Arksey and O’Malley’ framework [14]. However, few studies mentioned the dissemination of findings [22], which may be due to the optional nature in the Asksey and O’Malley framework.

In general, scoping reviews published in English demonstrated higher methodological and reporting quality compared to those published in Chinese. However, some key items recommended by JBI guidance were also poorly reported, including protocol registration, search strategy and data presentation. The significance of the protocol has been emphasized in both the JBI guidance and the PRISMA-ScR checklist (Peters et al., 2022). Surprisingly, less than 10% of the included scoping reviews in our study provided information about the protocol. It is worth noting that various platforms such as Figshare, Open Science Framework, ResearchGate, and Research Square allow protocol registration, and it is encouraged to include full protocols with available information for preregistration purposes.

Unsimilar to systematic review [23] and other evidence synthesis approaches, scoping reviews have the flexibility to include various types of literature including grey literature, newspapers, websites and social media, to address the question of “what has been done before” [24]. However, we found a limited number of included scoping reviews that conducted the additional search or consulted with information specialists. Furthermore, among those reviews performed database searches, only half of them provided a comprehensive search strategy for at least one database. It is important to consider conducting a more comprehensive search during the planning phase of a scoping review.

The scoping reviews included in our study showed a preference for presenting results in tables rather than images, resulting in a lack of diversity in data presentation. To enhance the interpretability of scoping review findings, various engaging methods, such as bubble charts, infographics, and Wordless, are available. We recommend that researchers, journal editors, and peer reviewers undergo additional training courses or access online resources (Stern et al., 2018) to improve the methodological quality of scoping reviews conducted by Chinese academic institutions. Furthermore, journal editors should require authors submitting scoping reviews to adhere to the PRISMA-ScR checklist before final submission. Additionally, we encourage research management agencies to promote the practice of conducting a scoping review in their respective fields prior to the initiation of research projects, helping researchers gain a better understanding of their research background and reduce research waste (Khalil et al., 2022).

Our findings are broadly consistent with the two previous scoping reviews of scoping reviews [7, 25], demonstrating considerable variability in the purpose, topics, and methodological aspects. However, our study finds certain improvements in specific areas, such as a better understanding of the distinctions between scoping review and systematic review [26–28], as well as the adoption of descriptive conclusions instead of definitive conclusions for practice.

To identify all relevant scoping reviews conducted by Chinese academic institutions, we conducted a comprehensive search that encompassed grey literature and web search engines. Based on our findings, we provide suggestions to researchers, journal editors, and administrators. However, our study also has some limitations. We may have partially missed some reviews conducted by Chinese academic institutions if the author’s name was not a traditional Chinese form. Additionally, we only included studies in Chinese and English, which may have excluded reviews published in other languages by authors from Chinese academic institutions.

## Conclusion

The annual number of scoping reviews conducted by Chinese academic institutions has shown a significant upward trend since 2020, encompassing a wide range of research topics, particularly within the realms of nursing and health. Scoping reviews are increasingly employed in practical applications, such as research preparation and identification of research questions. However, there remains a notable deficiency in the methodological rigor and reporting quality of scoping reviews conducted by Chinese academic institutions. Future research should prioritize enhancing the transparency of search and screening processes, diversifying data presentation techniques, and promoting standardization in reporting practices.

### Electronic supplementary material

Below is the link to the electronic supplementary material.

Supplementary Material 1

Supplementary Material 2

Supplementary Material 3

Supplementary Material 4

Supplementary Material 5

## Data Availability

The data used to support the findings of this study are included within the article and the supplementary information file.

## References

[1] Moher D, Stewart L, Shekelle P (2015). All in the family: systematic reviews, rapid reviews, scoping reviews, realist reviews, and more. Syst Rev Dec.

[2] Grant MJ, Booth A (2009). A typology of reviews: an analysis of 14 review types and associated methodologies. Health Info Libr J Jun.

[3] Sutton A, Clowes M, Preston L, Booth A (2019). Meeting the review family: exploring review types and associated information retrieval requirements. Health Info Libr J Sep.

[4] Colquhoun HL, Levac D, O’Brien KK (2014). Scoping reviews: time for clarity in definition, methods, and reporting. J Clin Epidemiol Dec.

[5] Levac D, Colquhoun H, O’Brien KK (2010). Scoping studies: advancing the methodology. Implement Sci Sep.

[6] Davis K, Drey N, Gould D (2009). What are scoping studies? A review of the nursing literature. Int J Nurs Stud Oct.

[7] Tricco AC, Lillie E, Zarin W (2016). A scoping review on the conduct and reporting of scoping reviews. BMC Med Res Methodol Feb.

[8] Munn Z, Peters MDJ, Stern C, Tufanaru C, McArthur A, Aromataris E (2018). Systematic review or scoping review? Guidance for authors when choosing between a systematic or scoping review approach. BMC Med Res Methodol Nov.

[9] G MDJPC, BS PMC, K H. D. P. Chapter 11: scoping reviews. The Joanna Briggs Institute. Accessed April 1, 2020. https://reviewersmanual.joannabriggs.org.

[10] Daudt HM, van Mossel C, Scott SJ (2013). Enhancing the scoping study methodology: a large, inter-professional team’s experience with Arksey and O’Malley’s framework. BMC Med Res Methodol.

[11] Gough D, Thomas J, Oliver S (2012). Clarifying differences between review designs and methods. Syst Rev Jun.

[12] Harms MC, Goodwin VA (2019). Scoping reviews. Physiotherapy Dec.

[13] Mays N, Popay RE. J. Studying the organization and delivery of health services: research methods. In: Fulop. N, Allen. P, Clarke. A, Black. N, eds. *Synthesising research evidence*. Routledge; 2001:194.

[14] Arksey H, O’Malley L (2005). Scoping studies: towards a methodological framework. Int J Soc Res Methodol.

[15] Peters MD, Godfrey CM, Khalil H, McInerney P, Parker D, Soares CB (2015). Guidance for conducting systematic scoping reviews. Int J Evid Based Healthc Sep.

[16] Peters MDJ, Marnie C, Tricco AC (2020). Updated methodological guidance for the conduct of scoping reviews. JBI Evid Synth Oct.

[17] Peters MDJ, Marnie C, Tricco AC (2021). Updated methodological guidance for the conduct of scoping reviews. JBI Evid Implement Mar.

[18] Tricco AC, Lillie E, Zarin W (2018). PRISMA Extension for scoping reviews (PRISMA-ScR): Checklist and Explanation. Ann Intern Med Oct.

[19] Xiyi W, Zhihong Y, Leiwen T (2019). An integrative review of scoping review applied in nursing iterature. Chin J Nurs.

[20] Mu F, Tang M, Guan Y (2022). Knowledge mapping of the Links between the gut microbiota and heart failure: a Scientometric Investigation (2006–2021). Front Cardiovasc Med.

[21] Lin Z, Ji X, Tian N, Gan Y, Ke L (2021). Mapping intellectual structure for the long non-coding RNA in Hepatocellular Carcinoma Development Research. Front Genet.

[22] Straus SE, Tetroe JM, Graham ID (2011). Knowledge translation is the use of knowledge in health care decision making. J Clin Epidemiol Jan.

[23] Higgins J, Thomas J, Chandler J et al. Cochrane Handbook for Systematic Reviews of Interventions. http://handbook.cochrane.org/.

[24] Khalil H, Peters MD, Tricco AC (2021). Conducting high quality scoping reviews-challenges and solutions. J Clin Epidemiol Feb.

[25] Pham MT, Rajić A, Greig JD, Sargeant JM, Papadopoulos A, McEwen SA (2014). A scoping review of scoping reviews: advancing the approach and enhancing the consistency. Res Synth Methods Dec.

[26] Qian J, Sun S, Wang M, Yu X (2022). Nonpharmacological pain management interventions in medical and surgical abortion: a scoping review. Int J Nurs Pract Apr.

[27] Wang Y, Wang Z, Liu G (2022). Application of Serious games in Health Care: scoping review and bibliometric analysis. Front Public Health.

[28] Huang L, Chang H, Peng X, Zhang F, Mo B, Liu Y (2022). Formally reporting incidents of workplace violence among nurses: a scoping review. J Nurs Manag Sep.

