# Supplementary material for: A scoping review on the methodological and reporting quality of scoping reviews in China
Source: BMC Med Res Methodol. 2024 Feb 22;24:45. doi: 10.1186/s12874-024-02172-y (PMC10882808; doi:10.1186/s12874-024-02172-y)
Supplement: Supplementary file 2 — Supplementary Material 2 [file 12874_2024_2172_MOESM2_ESM.docx]

**Appendix1. Search Strategy**

**PubMed:**

("scoping review"[Text Word] OR "scoping reviews"[Text Word] OR "scoping overview*"[Text Word] OR "scoping search*"[Text Word] OR "scoping study"[Text Word] OR "scoping studies"[Text Word] OR "scoping exercise*"[Text Word] OR "mapping review"[Text Word] OR "mapping reviews"[Text Word] OR (("mapped"[All Fields] OR "mapping"[All Fields] OR "mappings"[All Fields]) AND "overview*"[Text Word]) OR (("mapped"[All Fields] OR "mapping"[All Fields] OR "mappings"[All Fields]) AND "overview*"[Text Word]) OR "literature mapping"[Text Word]) AND ("hong kong"[Affiliation] OR "China"[Affiliation] OR "Taiwan"[Affiliation])

**EMbase:**

1. (scoping adj2 review).tw.
2. (scoping adj2 reviews).tw.
3. (scoping adj2 overview*).tw.
4. (scoping adj2 search*).tw.
5. (scoping adj2 study).tw.
6. (scoping adj2 studies).tw.
7. (scoping adj2 exercise*).tw.
8. (mapping adj2 review).tw.
9. (mapping adj2 reviews).tw.
10. (mapped adj2 review).tw.
11. (mapped adj2 reviews).tw.
12. (mapping adj2 overview*).tw.
13. (mapped adj2 overview*).tw.
14. (literature adj2 mapping).tw.
15. (correlates adj2 review).tw.
16. (correlates adj2 reviews).tw.
17. or/1-16
18. Hong Kong.in.
19. China.in.
20. Taiwan.in.
