# Supplementary figures and images for: A scoping review on the methodological and reporting quality of scoping reviews in China
Source: BMC Med Res Methodol. 2024 Feb 22;24:45. doi: 10.1186/s12874-024-02172-y (PMC10882808; doi:10.1186/s12874-024-02172-y)

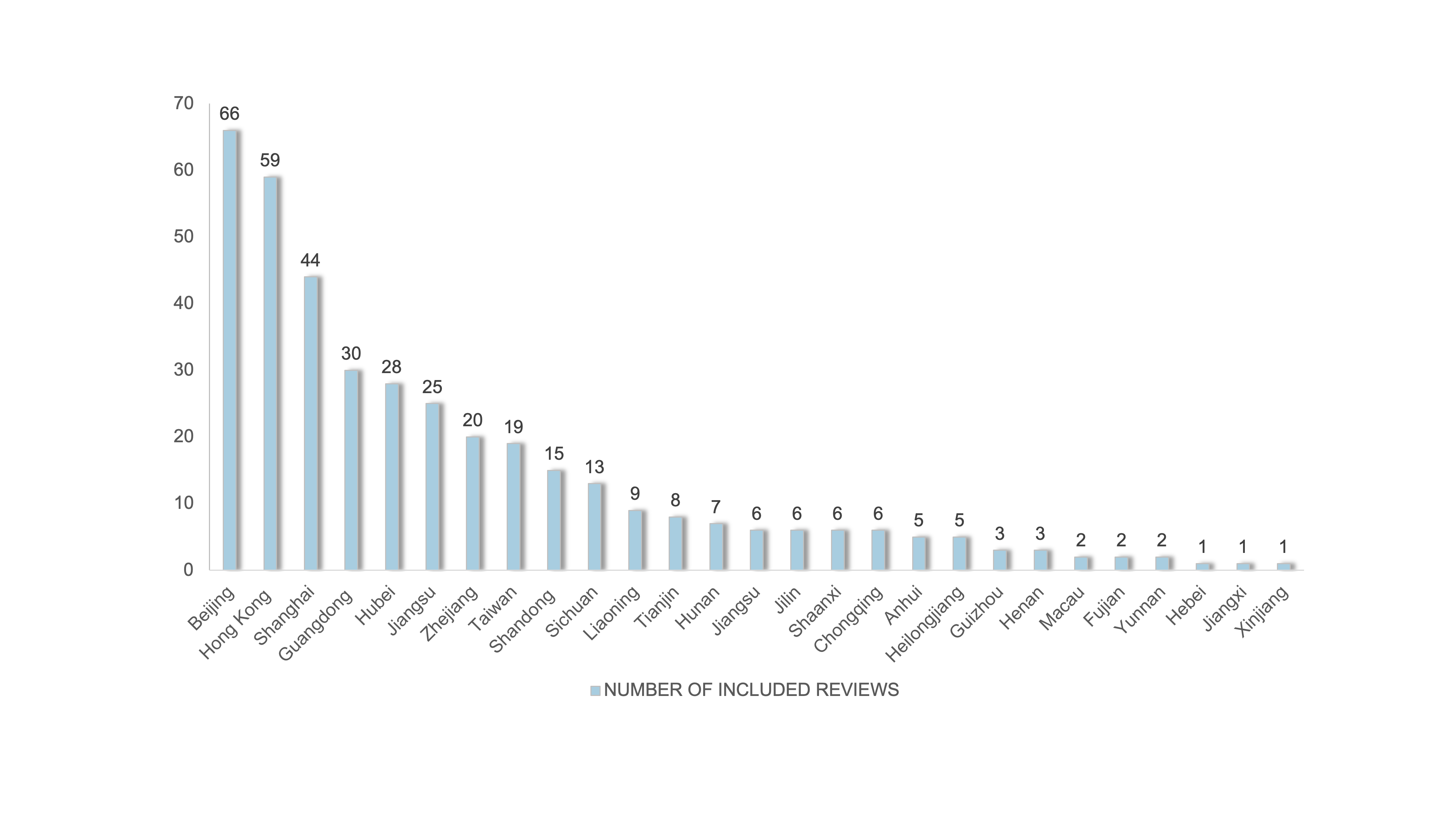

Supplement: Supplementary file 3 — Supplementary Material 3 [file 12874_2024_2172_MOESM3_ESM.tiff]

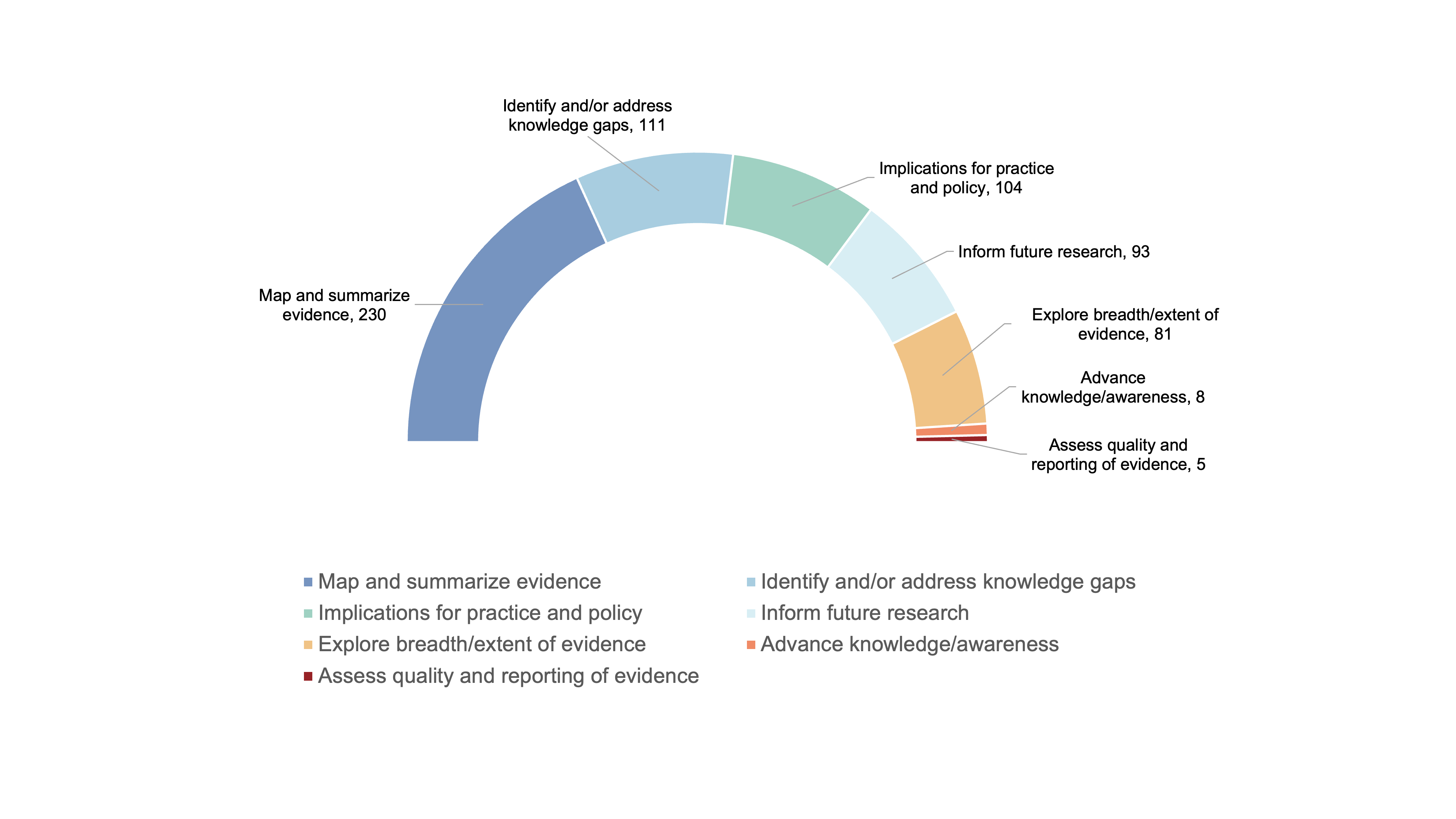

Supplement: Supplementary file 4 — Supplementary Material 4 [file 12874_2024_2172_MOESM4_ESM.tiff]

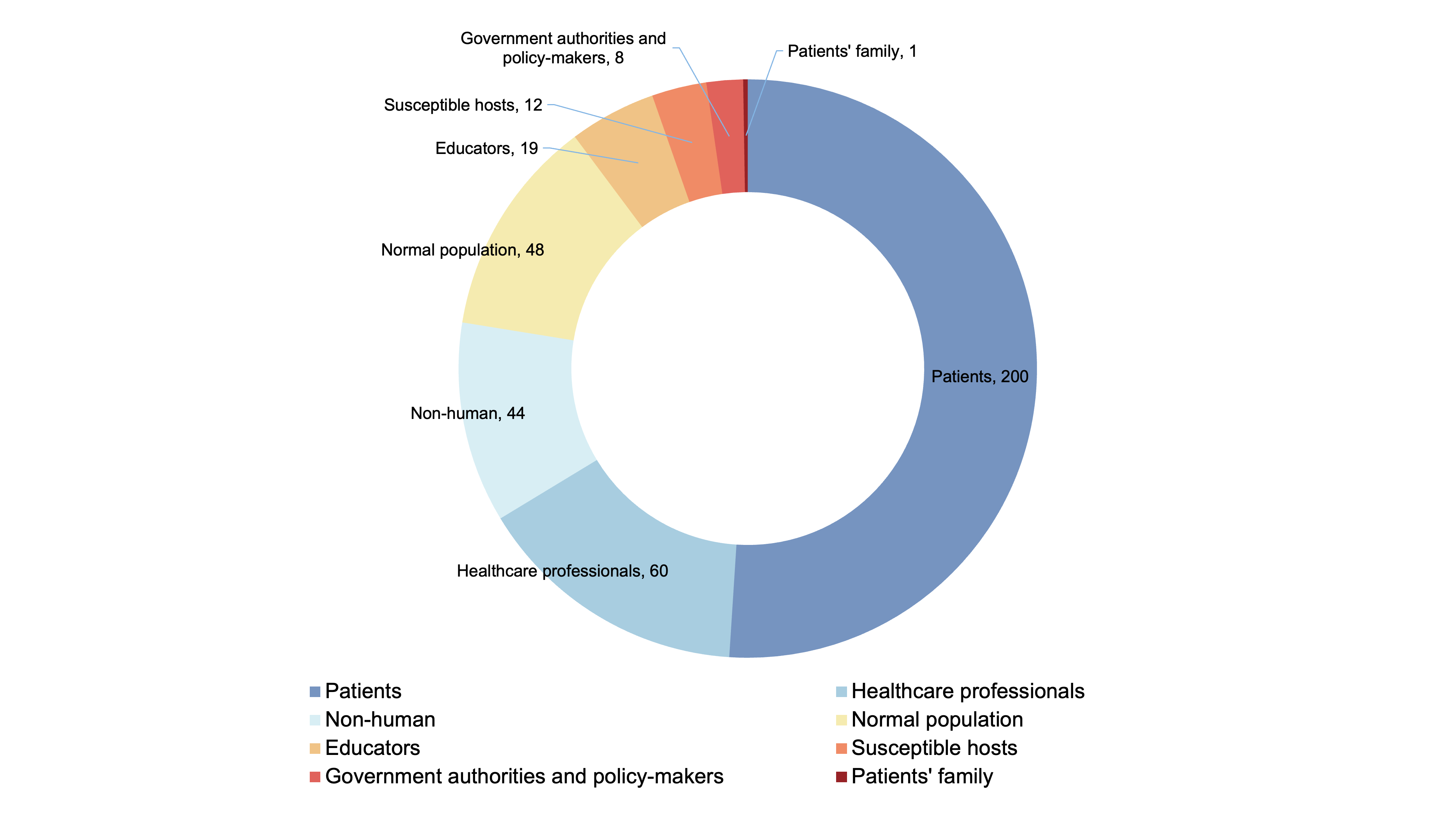

Supplement: Supplementary file 5 — Supplementary Material 5 [file 12874_2024_2172_MOESM5_ESM.tiff]
